# Development of an instrument to assess the impact of an enhanced experiential model on pharmacy students' learning opportunities, skills and attitudes: A retrospective comparative-experimentalist study

**DOI:** 10.1186/1472-6920-8-17

**Published:** 2008-04-08

**Authors:** Rosemin Kassam, Gary Poole, John B Collins

**Affiliations:** 1 Faculty of Pharmaceutical Sciences, The University of British Columbia, Vancouver, British Columbia, Canada; 2 Centre for Teaching and Academic Growth, The University of British Columbia, Vancouver, British Columbia, Canada; 3 Department of Educational Studies, The University of British Columbia, Vancouver, British Columbia, Canada

## Abstract

**Background:**

Pharmacy schools across North America have been charged to ensure their students are adequately skilled in the principles and practices of pharmaceutical care. Despite this mandate, a large percentage of students experience insufficient opportunities to practice the activities, tasks and processes essential to pharmaceutical care. The objective of this retrospective study of pharmacy students was to: (1) as "proof of concept", test the overall educational impact of an enhanced advanced pharmacy practice experiential (APPE) model on student competencies; (2) develop an instrument to measure students' and preceptors' experiences; and (3) assess the psychometric properties of the instrument.

**Methods:**

A comparative-experimental design, using student and preceptor surveys, was used to evaluate the impact of the enhanced community-based APPE over the traditional APPE model. The study was grounded in a 5-stage learning model: (1) an enhanced learning climate leads to (2) better utilization of learning opportunities, including (3) more frequent student/patient consultation, then to (4) improved skills acquisition, thence to (5) more favorable attitudes toward pharmaceutical care practice. The intervention included a one-day preceptor workshop, a comprehensive on-site student orientation and extending the experience from two four-week experiences in different pharmacies to one eight-week in one pharmacy.

**Results:**

The 35 student and 38 preceptor survey results favored the enhanced model; with students conducting many more patient consultations and reporting greater skills improvement. In addition, the student self-assessment suggested changes in attitudes favoring pharmaceutical care principles. Psychometric testing showed the instrument to be sensitive, valid and reliable in ascertaining differences between the enhanced and traditional arms.

**Conclusion:**

The enhanced experiential model positively affects learning opportunities and competency acquisition, as measured by a new instrument showing sound psychometric properties.

## Background

Preventable drug-related problems due to inappropriate prescribing, under-prescribing, and inappropriate medication use contribute significantly to both the economic and human costs of health care [1-4]. Several studies have demonstrated that pharmacists have a vital role to play in drug therapy management, thus enhancing overall patient health outcomes [5-10]. In response to economic and societal needs, the pharmacy profession throughout North America has adopted *pharmaceutical care *as its new practice mandate. Pharmaceutical care is defined as a philosophy of practice wherein the "pharmacist cooperates with patients and other professionals in designing, implementing and monitoring therapeutic plans that will produce specific therapeutic outcomes" [1]. Pharmaceutical care takes the profession beyond simple drug distributing and counseling responsibilities to a broader mandate of patient-centered care to maximize the positive outcomes of patients' drug therapies. Consequently, the Canadian Council for Accreditation of Pharmacy Programs has charged pharmacy schools across Canada to ensure that students are adequately skilled in the principles and practices of pharmaceutical care [11].

In 1999, the structured practice education program (SPEP) at the Faculty of Pharmaceutical Sciences (the Faculty), University of British Columbia (UBC) – Vancouver, Canada, used an iterative process involving faculty members and UBC pharmacy preceptors to introduce a new community-based advanced pharmacy practice experience (APPE) to meet accreditation standards. The new APPE detailed 13 content domains that emphasized the specific knowledge, skills and attitudes required to practice pharmaceutical care; these domains can be summarized under four general themes: (1) developing care delivery strategies that promote discussing, acquiring and assessing relevant patient, drug and disease; (2) developing care plans in collaboration with patients and other health professionals to prevent or resolve drug-related problems; (3) assuming responsibility for managing drug-related problems by monitoring patients' progress and ensuring follow-up care; and (4) promoting health through disease prevention and wellness clinics and seminars. Table 1 summarizes the range of specific patient care activities students are required to engage in for this APPE. The development process of this new program has been discussed in detail in a previous publication [12].

**Table 1:** Community-based advanced pharmacy practice experience activities

**PC Competency Domains**		**Activity Description**
1. Asking about patient expectations		1. Assess patients with new prescriptions and develop care plans to resolve/prevent drug-related problems
2. Collecting relevant information		2. Assess patients with refill prescriptions and develop care plans to resolve/prevent drug-related problems
3. Integrating patient information		3. Present and discuss 1 prescription AND 1 non-prescription drug class with preceptor
4. Evaluating different treatment options		4. Provide pharmaceutical care patients requesting non-prescription products, develop care plan for all interventions
5. Documenting patient info: continuity of care		5. Provide follow-up to patients encountered in activities # 1, 2, 4 and 9, document follow-up care
6. Prioritizing drug related problems		6. Provide drug information to patients, preceptors and other health care providers, document all recommendations
7. Determining patient experiences: effectiveness or undesirable effects of current medications		7. Shadow another health care professional for 1/2 to 1 day, complete the reflection page
8. Determining whether patients were managing and adhering to their medication regimes		8. Discuss pharmacy practice issues related to pharmaceutical care (barriers and opportunities
9. Establishing monitoring parameters with patients		9. Provide comprehensive pharmaceutical care by assessing all drug-related needs of your patient, identify drug-related problems and develop care plans to resolve/prevent drug-related problems
10. Following-up patients by phone or in-person		10. Initiate and complete a patient care project, submit a summary of your project
11. Developing professional relationships: with other health care providers, physicians		
12. Participating in clinics, seminars, projects or presentations		
13. Providing basic and comprehensive pharmaceutical care		

As part of the SPEP office's continuous quality improvement initiative, a detailed evaluation of the newly implemented community-based APPE was conducted in 2000 [13,14]. The findings from the evaluation suggested that – while students had ample opportunity to engage in learning activities that the pharmacy staff regularly encountered in their practices, such as assessment of new and refill prescriptions and over-the-counter requests, they had significantly fewer opportunities to engage in activities related to comprehensive pharmaceutical care that included developing and maintaining relationships with patients, assuming responsibility for the management of drug-related issues, and evaluating the patients' drug therapy through follow-up care. Student learning was further hampered by limited opportunities to engage in a variety of non-direct pharmaceutical care activities, such as interprofessional collaborations and health promotion/disease prevention clinics, seminars and presentations. Interestingly, results from a recent survey found that a large percentage of students from the United States also experienced insufficient opportunities to practice the activities, tasks and processes essential to pharmaceutical care; suggesting this to be a widespread concern [15].

To enhance UBC students' learning experience during their community-based APPE, the SPEP faculty undertook a project to explore structures and processes that could help promote better congruence with the principles of pharmaceutical care and the patient care delivery process the students engaged in. The structures and processes were introduced in a stepwise fashion, supported by evidence gathered at different stages of the project. This article reports on a pilot study focusing on three goals: (1) as "proof of concept," to test the overall educational impact of an enhanced APPE model on student competencies; (2) to develop an instrument to measure how both students and preceptors experienced the enhanced community-based model; and (3) to assess the reliability, validity and discriminating power of the instrument to detect differences between the traditional and the enhanced APPE models. The primary hypothesis was that the competency scores for the enhanced model (treatment arm) would improve more than would scores for the traditional model (control arms), and that such improvements would be evident for both students and their preceptors.

## Methods

### Design

A retrospective comparative-experimentalist design was used to evaluate the impact of the enhanced community-based APPE model on students' learning and performance [16]. The overall logic of the study was grounded in a 5-stage learning model: (1) An enhanced learning climate (trained preceptors, explicit SPEP guidelines, student training and support) should lead to (2) better utilization of APPE learning opportunities, including (3) more frequent student/patient consultation, then to (4) improved skills acquisition, thence to (5) more favorable attitudes toward pharmaceutical care practice. The study was conducted between September 2001 and May 2002 in community pharmacies with continuing histories as placement sites for APPE. Ethics approval had been obtained through UBC's Office of Research Services and consent was obtained from all students and preceptors to allow dissemination and publication of the study's findings.

### Participants

This study was grounded in a unique partnership developed between the University's Pharmacy school and a nationally operating pharmacy chain. As part of this partnership, the national chain had agreed to two important aspects: (1) to provide release time for their preceptors to attend a full-day's preceptor training workshop at company expense; and (2) to support the costs associated with the delivery of the training program and the evaluation of the APPE post-training. A student researcher was hired to manage data collection and collation, and an instrument developer/statistician was hired to assist with survey instrument development, data analysis and interpretation.

The aim of the study was to recruit seven community pharmacies into the study's treatment arm (enhanced rotation) and a similar number into each of the two control arms (traditional rotation). To extend the generalizability of the study results, one of the control arms comprised pharmacies from the same national partnering chain while the second control arm consisted of an assortment of community pharmacies from other chains and independent pharmacies. Purposeful selection was used to recruit the pharmacies, ensuring representation from both urban and rural settings; all sites had a long history of serving as placement sites for UBC. The community pharmacies in the two control arms were matched to the enhanced pharmacies in terms of geographic location and community size, and all had previously served as placement sites with UBC. All recruited pharmacies were asked to designate one pharmacist to serve as the primary preceptor, whose responsibility would be to set the clerkship expectations with the student, facilitate learning opportunities, provide on-going feedback and conduct the mid-point and final evaluation of the student. In addition, other pharmacists were also encouraged to participate in the student's learning if they were interested in doing so; these pharmacists served as secondary preceptors and worked within the framework established by the primary preceptor. Student participation in the enhanced arm was voluntary because its structure was new to the curriculum, would require additional student commitment beyond the traditional rotation, and its impact had not yet been assessed. The enhanced APPE was advertised to all fourth-year (senior year) students through e-mail and class presentations, and interested students were recruited on a first-come basis. Each placement site (enhanced and control) accepted a maximum of two students at different times within the academic year.

### Intervention

During their 8-week community-based APPE, students in both the enhanced and control sites were expected to meet similar learning objectives, participate in the same learning activities, and were held to the same learning and performance criteria and evaluation standards.

Students and preceptors in both control arms received the SPEP manual outlining the APPE's learning activities, expectations, policies, evaluation processes and forms, patient care tools and conduct standards. The APPE duration for the control arm students comprised of two 4-week cycles at two different pharmacy locations. The control students participated in a mandatory 3-hour face-to-face orientation session and an on-line quiz reinforcing the APPE expectations, but no additional intervention was provided to the control preceptors other than the routine verbal and written communications that occurs between the SPEP office and the preceptors.

Primary preceptors and students in the enhanced arm were provided with a few additional interventions beyond those offered to the control arm. The intervention consisted of separately structured experiences for both students and preceptors: (1) a one-day preceptor education workshop to discuss the proposed pharmaceutical care practice model, to review the course syllabus, to clarify the learning tasks expected of students, and to work through an ice-breaker exercise to be conducted with their students; (2) a five-day student orientation at the pharmacy site prior to the start of the practice education experience to allow pharmacists to assess their students' baseline competencies with various distribution activities and selected pharmaceutical care activities outlined in Table 1 (specifically activities: # 1, 2, 4, 6 and 9); and (3) extending the experience from the traditional two 4-week rotations completed at two different pharmacies to one 8-week experience completed at the same enhanced arm pharmacy.

### Instrument Development

A 70-item, 5-point Likert-type survey was developed around the pharmaceutical care activities and competencies students were expected to master. The survey layout recapitulated the 5-stage learning model: [1] learning climate and preceptor support (9 questions), [2] learning opportunities (13 questions), [3] patient consultation estimates (one question), [4] skills improvement (17 questions), and [5] attitude enhancement (29 questions). In approximate terms, the survey was structured around the 13 pharmaceutical care competency domains emphasized in the community-based APPE (Table 1). In general, each of the competency domains was assessed three times; once as a learning opportunity issue, secondly as a skill improvement topic, and third as an attitude-related matter. A final question for preceptors alone asked whether the experience of precepting students had expanded their own grasp of pharmaceutical care principles and practice.

Instrument development activities focused on verifying that questionnaire items putatively assigned to preceptor supports scales, learning opportunities, skills improvement, and attitudes all conformed to generally accepted scale development criteria, in that they: (1) distinguished effectively between enhanced and control settings; (2) demonstrated alpha reliabilities equal to or exceeding the rule-of-thumb threshold of α = 0.70; (3) converged reliably on their respective specific themes; (4) did not "spill across" to other themes; and (5) gathered information unbiased with respect to source (preceptor or student). Scales and items passing all these tests would be retained for further refinement in post-pilot refinements and future iterations of the enhanced APPE model [17,18].

### Data collection

Both the students and their primary preceptors completed retrospective surveys shortly following the completion of the APPE. As well, both groups were assured that survey results would not affect the students' final grades. The survey instructions requested the preceptors in the enhanced group and students from both groups to reflect back to the baseline day of their 8-week APPE. The control preceptors assessed the students over the second 4-week experience. While both the students and preceptors were asked about virtually identical topics, the question phrasing was keyed to the appropriate audience, for example: student items were phrased in terms of "There were opportunities to ...", "I believe that..." whereas preceptor items were phrased in terms of "The rotation provided the student with...", or "I met with the student to...". Each survey packet consisted of seven generic Scantron sheets (#70921) overprinted with instructions, question stems and 5-point response categories; 'Never' to 'Always', 'No Improvement' to 'Significant Improvement' or 'Strongly Disagree' to 'Strongly Agree'. Surveys were distributed to students and preceptors by mail, and returned either by mail or in-person.

### Analysis

Scantron survey results were verified, summarized and analyzed using SPSS. Results of the study's outcomes progressed though a series of increasingly stringent analyses; overall summaries (SPSS Frequencies and Descriptives), differences between enhanced and control arms (t-tests, ANOVA), differences between preceptor and student assessments (t-tests, ANOVA) and possible interactions (ANOVA), reliability and validity estimates of competency domains and study rationale measures (Reliability), and tests of the various scales' abilities to discriminate between enhanced and control conditions (Discriminant). For all tests, the a priori level of significance was set at p ≤ 0.05.

Since the overall objective of the pilot study was to test the impact on student learning of the enhanced APPE, we report those overall results first, followed by more detailed analyses of the learning model components (learning climate and preceptor support → learning opportunities + patient consultations → skills improvement → attitude enhancement). Next we report differences across the 13 domains of pharmaceutical care and the differential impact of learning in the enhanced and control settings. Finally, we report the psychometric properties of the scales we developed to examine these various aspects of program enhancement and student performance with a focus on scale refining and streamlining. We conclude with a number of 'lessons learned' about further improvements to the APPE, easier data collection procedures and expanded scope of future enhanced APPE.

## Results

At the end of the APPE, there were 13 students who completed the enhanced APPE (treatment arm) and 28 students in the two control APPEs (14 in each arm), one student from the enhanced arm dropped out prior to the start of the APPE. All sites had one designated primary preceptor, two sites in each of the three arms had a secondary preceptor, one site in the treatment arm had two secondary preceptors. A total of 74 surveys were returned at the end of the study: 38 from preceptors and 36 from students. From the preceptor group, 13 (100%) surveys were returned from the enhanced arm and 11 (78.6%) from the control arm representing the national partnering chain and 14 (100%) from the control arm representing an assortment of pharmacies. From the student group, 13 (100%) surveys were returned from the enhanced arm, nine (64.3%) from the national chain control arm and 14 (100%) from the control arm representing an assortment of pharmacies. One student from the control arm left substantial portions of the survey unanswered and was eliminated from further analysis, for a total of 35 useable student surveys.

### Overall Results

Table 2 demonstrates that, overall, student and preceptor outcomes favored the enhanced APPE structure over the structure in the control arms. From the students' own perspectives and for all 70 measures combined, student scores in the enhanced APPE outperformed those in the control arm by about 0.37 points out of a possible five: (4.16 vs. 3.79; t = 2.33, p < 0.026). Parallel information from preceptors about their students' overall performance generally corroborated students' own assessments. For all 70 measures combined, preceptors' assessments showed a significant 0.39 point advantage for the enhanced APPE, (4.07 vs. 3.68; t = 2.67, p < 0.011) slightly exceeding students' own reports. From students' and preceptors' combined perspectives, the enhanced APPE showed similar statistical advantages; the enhanced APPE outperformed the experience in the control arm by 0.38 points (4.11 vs. 3.73; t = 3.59, df = 71, p < 0.001) and showed an overall 18% gain in performance (η^2 ^= 0.179).

**Table 2:** Student and preceptor results for the 5-component learning model

**Perspective**	**Group**	**Mean**	**t-test**	**Significance**
**Learning Climate and Preceptor Supports **(number (n) of items = 9; score range: 2.00 – 5.00)				

Students'	Control APPE	3.87	1.21	ns
	Enhanced APPE	4.21		
Preceptors'	Control APPE	3.73	1.89	ns
	Enhanced APPE	4.07		

**Learning Opportunities **(n items = 13; score range: 2.46 – 4.62)				

Students'	Control APPE	3.76	0.34	ns
	Enhanced APPE	3.82		
Preceptors'	Control APPE	3.50	1.56	ns
	Enhanced APPE	3.79		

**Estimated Number of Comprehensive Consultations **(n items = 1; score range: 1.00 – 17.00)				

Students'	Control t APPE	5.30	7.29	p < 0.000
	Enhanced APPE	16.65		
Preceptors'	Control APPE	8.74	5.65	p < 0.000
	Enhanced APPE	17.50		

**Skills Improvement **(n items = 17; score range: 1.94 – 5.00)				

Students'	Control APPE	3.50	3.05	p < 0.004
	Enhanced APPE	4.21		
Preceptors'	Control APPE	3.22	3.81	p < 0.001
	Enhanced APPE	4.12		

**Attitudes **(n items = 29; score range: 2.90 – 5.00)				

Students'	Control APPE	4.03	2.41	p < 0.021
	Enhanced APPE	4.39		
Preceptors'	Control APPE	4.16	0.86	ns
	Enhanced APPE	4.31		

**Overall Scale **(n items = 70; score range: 2.65 – 4.89)				

Students'	Control APPE	3.78	2.33	p < 0.026
	Enhanced APPE	4.16		
Preceptors'	Control APPE	3.68	2.67	p < 0.011
	Enhanced APPE	4.07		

### The 5-Stage Learning Model

Table 2 also summarizes results for each of the five stages of the learning model: learning climate and supports, learning opportunities, patient consultations, skills improvements, and pharmaceutical care-related attitudes. The results always favored the enhanced APPE model, with two-thirds of the comparisons being statistically significant. Since ONEWAY Tukey range tests had shown there were no statistical differences between the two control groups for either students or preceptors, the two control arms were collapsed into a single control group for further analyses.

Students reported statistically significant benefits of the enhanced APPE in terms of the number of comprehensive consultations it afforded (16.65 vs. 5.30; t = 7.29, p < 0.000), skills improvements noted (4.21 vs. 3.50; t = 3.05, p < 0.004) and attitudes favoring pharmaceutical care principles (4.39 vs. 4.03; t = 2.41, p < 0.021). Preceptors in the enhanced model reported noted benefits of the enhanced APPE, the estimated numbers of comprehensive consultations conducted by students (17.50 vs. 8.74; t = 5.65, p < 0.000), and skills improvement observed (4.12 vs. 3.22; t = 3.81, p < 0.004). Although more preceptors in the enhanced model agreed that their SPEP participation "provided [more] opportunity to practice and improve my pharmaceutical care skills" than did preceptors in the control arms, this was not statistically significant.

It is also noteworthy that a two-way ANOVA showed generally no differences between students' self-reports and those of their preceptors, except for the finding that preceptors in both the control and enhanced arms significantly (F = 5.65, p < 0.020) over-estimated the number of patient consultations (11.7 vs. 9.5) provided by their students (and verified by the SPEP faculty by reviewing documentation submitted by the students in their portfolio). Interestingly, the overestimates were greater in the control settings than in the enhanced APPE, perhaps suggesting that preceptors' oversight of student experience is less detailed in controlled settings. As well, there were no treatment-by-group interactions indicating that both students and preceptors interpreted and responded to the questions in essentially the same ways.

### Pharmaceutical Care Competency Domains

Table 3 shows that some competencies were much better learned than others – irrespective of study arm. For ease of interpretation, the central columns of the table shows competency means converted to percentages (mean/5) for overall results as well as for enhanced/control differences, thus showing the fraction of what was reported to have been learned as differential percentages of the maximum that could be reported. Examining all groups combined (enhanced, control, students, and preceptors), the table shows that content domains differ considerably in their respective levels of learning achievement (M%); ranging from lows of about 40% to highs of near 90%. For example, topics such as *collecting relevant patient information *(4.38 out of 5) or *determining whether patients were managing their medication regimes *(4.17) were readily grasped while *participating in clinics, seminars, projects or presentations *(1.99) or *developing professional relationships with other health-care providers *(3.47) *w*ere much less well inculcated. Finally, the bottom row of Table 3 summarizes the overall student grasp of these 13 competencies and suggests that about 77% of what could have been reported was reported. Moreover, it shows that the enhanced rotations enjoyed about a 7% advantage over the traditional model (F = 11.40, p < 0.001) in bringing students nearer to readiness for entry-to-practice.

**Table 3:** Competency domains for learning pharmaceutical care principles and delivery:Overall, enhanced (treatment) and traditional (control) scores for students and preceptors combined.

**PC Competency Domains**	**Overall Mean**	**Overall Mean %**	**Enhanced Mean %**	**Control Mean %**	**Significance**^a^
Asking about patient expectations	3.84	76.9	82.1	74.0	0.005
Collecting relevant information	4.38	87.6	91.3	85.5	0.036
Integrating patient information	4.08	81.6	89.0	77.6	0.000
Evaluating different treatment options	4.18	82.5	87.4	79.7	0.010
Documenting patient info: continuity of care	3.91	78.3	85.4	74.3	0.001
Prioritizing drug related problems	3.97	79.4	84.6	76.5	0.002
Determining patient experiences: effectiveness or undesirable effects of current medications	4.23	84.6	88.5	82.4	0.023
Determining whether patients were managing and adhering to their medication regimes	4.17	83.4	87.2	81.3	0.022
Establishing monitoring parameters with patients	3.95	78.9	86.2	74.9	0.000
Following-up patients by phone or in-person	3.90	77.9	85.8	73.6	0.000
Developing professional relationships: with other health care providers, physicians	3.47	69.5	69.2	69.6	ns
Participating in clinics, seminars, projects or presentations	1.99	39.9	37.6	41.1	ns
Providing basic and comprehensive pharmaceutical care	4.14	82.7	88.0	79.8	0.001
Overall Index of Content Domain Learning	3.86	77.2	81.7	74.6	0.001

^a ^Indicates significance of the differences observed between the enhanced (treatment) versus traditional (control)

When comparing the students' experiences between the enhanced and the control arms for all 13 of the competency domains, the outcomes consistently favoured the enhanced model; with six of the 13 competencies being significantly improved. These included competencies: "Integrating patient information," "Documenting patient information," "Prioritizing drug-related problems," "Establishing monitoring parameters with patients," " Patient follow-up by phone or in-person," and "Providing basic and comprehensive pharmaceutical care." Additionally, the overall index of learning in all 13 pharmaceutical care competency domains was significantly better for students in the enhanced arm (4.13 vs. 3.81; t = 2.15, p < 0.038) than the control arm – an improvement of 6.25%.

The preceptor pattern was slightly different; they reported significant improvement in seven of the 13 competencies: "Asking about patient expectations," "Collecting relevant information," "Integrating patient information," "Documenting patient information," "Establishing monitoring parameters," "Patient follow-up by phone or in-person," "Providing basic and comprehensive pharmaceutical care," as well as in the overall index of competency domains (4.04 vs. 3.66; t = 2.61, p < 0.013).

### Instrument Validation

Tables 4 and 5 summarize the broad array of analyses conducted to validate and test the survey instrument. The accepted criteria of face validity, reliability, discriminant validity, construct validity, absence of bias, and sensitivity were all used as benchmarks to determine whether the survey's items, scales, and psychometric properties achieved acceptable levels.

**Table 4:** Alpha-reliability coefficients, convergent and discriminant estimates for original study scales.

**Learning Model: Original Scales**	Mean*	**Standard Deviation**	**Number of Items**	**α^a^**	**r^b^**	**Scale Bias^c^**	**Scale Sensitivity^d^**
Learning Climate & Preceptor Support	3.92	0.69	9	0.884	0.549	0.110	0.305
Learning Opportunities	3.69	0.55	17	0.884	0.574	0.162	0.180
Patient Consultations (estimated number)	10.67	6.66	1	0.475e	0.475	-0.0168	0.520
Skills Improvement	3.68	0.73	17	0.938	0.476	0.118	0.510
Attitude Enhancement	4.19	0.46	29	0.939	0.479	-0.052	0.261
Overall Study Model Scale (original)	3.87	0.47	70	0.959	0.816	0.121	0.410

α^a ^– Cronbach's alpha-reliability, and the index of convergent validity; how well do items converge on each scale's central concept.

r^b ^– Highest correlation with any other scale. Low correlations mean the scale does not duplicate information in other scales.

^c ^– Pearson r with preceptor vs. student. Non-significant r's indicate absence of bias.

^d ^– Pearson r (or eta) with study arm. Positive correlations indicate sensitivity to predicting enhanced clerkship.

^e ^– Since the patient consultation estimate was a single variable measure, its α-reliability estimate is its SMR with the other four indices.

**Table 5:** Psychometric properties of revised learning model scales.

Learning Model: Refined Scales	Mean	Standard Deviation	Number of Items	α^a^	r^b^	Scale Bias^c^	Scale Sensitivity^d^
Preceptor Support	3.94	0.69	6	0.847	0.510	0.104	0.336
Learning Opportunities	4.00	0.62	6	0.837	0.510	0.214	0.304
Patient Consultations (estimated number)	10.67	6.66	1	0.475	0.580	-0.182	0.715
Skills Improvement	3.65	0.86	6	0.873	0.520	0.093	0.622
Attitude Enhancement	4.03	0.65	6	0.837	0.520	-0.157	0.393
Overall Study Model Scale (refined^f^)	3.89	0.56	25	0.920	0.796	0.057	0.574
Overall Study Model Scale (original)f	3.87	0.47	70	0.959	0.816	0.121	0.410

α^a ^– Cronbach's alpha-reliability, and the index of convergent validity; how well do items converge on each scale's central concept.

r^b ^– Highest correlation with any other scale. Low correlations mean the scale does not duplicate information in other scales.

^c ^– Pearson r with preceptor vs. student. Non-significant r's indicate absence of bias.

^d ^– Pearson r (or eta) with study arm. Positive correlations indicate sensitivity to predicting enhanced clerkship.

^e ^– Since the patient consultation estimate was a single variable measure, its α-reliability estimate is its SMR with the other four indices.

^f ^– Both Refined and Original scales are listed to document that streamlining resulted in no appreciable loss of scale power and efficiency.

#### Face validity

Face validity tests whether the individual items in any instrument "make sense" and whether knowledgeable experts concur that they are "important things to be asked". The instrument's 70 items were reviewed and verified by several cycles of item generation, pre-testing and refinement by this report's first two authors. The guiding question was always "Is this what we want to know about student response to the two versions of the APPE, and is this the way to ask it?" Subsequently, five faculty and five randomly selected non-study pharmacy students reviewed the survey items and provided helpful comments on readability and clarity.

#### Reliability

Reliability analysis examines whether the various items thought to constitute a scale are well-aligned, consistent among themselves, and generally corroborate each other. Contrary items can be refined, rephrased, or eliminated, although overall reliability measures depend on the number of items in the scale. The most common index of scale reliability is Cronbach's alpha (α) and scale developers strive for reliability measures of 0.70 or greater. Table 4's fourth column (α^a^) shows that four of the five learning model scales (preceptor support (0.88), learning opportunities (0.88), skills improvement (0.94) and attitude enhancement (0.94) far exceed the guideline, indicating that the individual items within each scale are good operational measures of the scale's conceptual content while simultaneously lending strong support to the construct validity of the scales' conceptual foundations. Since patient consultations is a single measure count, the concept of scale reliability doesn't apply in the same way, hence the table entry is its squared multiple correlation (0.48) with the four remaining scales. In short, the various scales comprising the 5-step learning model are well operationalized by the items constituting them.

For the 13 pharmaceutical care competency domains, their reliabilities are acceptable but not quite so strong because the number of items in most scales is often only three or four. For example: "following-up ...(0.76)," "developing professional relationships ...(0.77)," "participating in clinics ...(0.78)," and "providing basic and comprehensive pharmaceutical care ...(0.84)" are well operationalized. In contrast, " asking about patient expectations ...(0.27)," "prioritizing about drug-related problems ...(0.33)" and "determining if patients are managing/adhering...(0.46)" are less well-anchored. Nevertheless, the reliability of the overall index calculated across all 13 domains is 0.92, strong by any measure. Thus the scales derived from the 70-item survey generally exhibit reliabilities that are "acceptable" to "strong."

#### Discriminant validity

Discriminant validity analysis tests whether the items in any one scale are all more closely correlated among themselves than with any different scale, in short, do any of the items "spill over" into the domain of a different scale? Table 4, column r^(b) ^show that the highest correlations between any one scale in the learning model and any other scale are always lower than their respective alpha reliabilities indicating that each scale converges on its central concept and discriminates it from the other scales [18].

#### Construct Validity

Construct validity is a 'concept in reverse' [19]. Rather than querying how well each scale (or construct) is operationalized, it tests the proposition that "given the operational evidence, how much credibility can be (backwards) ascribed to the construct (or scale). If the operational evidence is strong, then there are more solid grounds to accept the notion that "there *is* such as thing as learning climate, or learning opportunity, or skills improvement or attitude enhancement". In this study, the learning climate total (of 9 items), learning opportunities total (of 17 items), patient consultation estimate, skills improvements total (of 17 items), attitude enhancement total (of 29 items), and the combined study model total (of 70 items) all have strong construct validities as evidenced by their high corrected item-total correlations (not shown) and their high α-reliabilities (0.85 and higher). Collectively, these reliability measures and item-total correlations confirm that such concepts as learning climate, learning opportunities, skills improvement and attitude enhancement *do* exist and can be validly inferred from the response patterns of pharmacy students and their preceptors.

#### Scale bias

Tests for scale bias seek to verify that both individual items and the scales constructed from them are unbiased in terms of the respondent who happens to be completing the survey. In this study, it meant testing that both students and preceptors understood and interpreted the items in essentially the same way. Study results would be weakened if it could be shown that any of the learning model scales favoured preceptors over students or vice-versa. The 'scale bias' column in Table 5 shows that correlations between scale totals and the persons completing the surveys (students vs. preceptors) are small and non-significant, hence threats of study outcomes being spurious results of student/preceptor differences can be ruled out. Further evidence that preceptors and students saw the items and scales similarly was obtained by correlating student/preceptor pairs' responses across the various practice locations. In general, preceptor/student pairs concur about the learning aspects of the APPE: the correlation between the pairs for combined feature of the overall learning model was 0.472. The overall correlations across all components of the model are fractionally higher for the enhanced pairs (0.495) than for the controls pairs (0.460), thus confirming that there was general, if loose, agreement between preceptors and students about when Learning Opportunities were (or were not) available, when clinical instructors had (or had not) facilitated in such as way as to enable students to meet their overall SPEP objectives, when Skills Improvements had (or had not) occurred, and when students' Attitudes and Attributions of Importance favoured (or did not favour) pharmaceutical care focused practice.

#### Scale Sensitivity

To be helpful in testing for differences between enhanced and control APPE, both survey items and the scales derived from them must be sensitive to any differences that do exist. The rightmost column in Table 4 shows that the estimate of numbers of patient consultations performed was the most sensitive discriminator between people in enhanced APPE vs. those in control settings. Skills improvement, learning climate, attitude enhancement and learning opportunities showed decreasing sensitivity to enhanced/control differences. Some 19 of the 70 were significant and sensitive indicators of enhanced vs. control differences: 11 from Skills Improvement, five from Attitude Enhancement, two from Learning Climate and Number of patient consultations. Disregarding for the moment the scales into which the 70 items were aggregated, in descending order of sensitivity, the 19 best discriminating items were: number of patient consultations, patient follow-up, integrating information from patients with multiple problems, providing comprehensive pharmaceutical care to patients with few problems, integrating patient information to identify drug-related problems, prioritizing drug-related problems, determining patient experiences regarding drug effectiveness and undesirable effects, evaluating different treatment options using guidelines, *dis*affirming that follow-up is too time-consuming, discussing monitoring parameters with patients, asking patients about their drug therapy expectations, determining whether patients were managing their medications, carrying out independent tasks without undue reliance on the pharmacist, preceptors encouraging students to provide patient follow-up, believing that providing pharmaceutical care gave students a better understanding of patients drug-related needs, acknowledging that the SPEP rotation was a key factor in stimulating pharmaceutical care, feeling comfortable with the process of providing pharmaceutical care, agreeing that discussing monitoring parameters with patients is important, and *dis*agreeing that providing patient follow-up is beyond a pharmacist's responsibility.

### Learning Model Scale Inter-Correlation

Correlations among the scales for learning climate, learning opportunities, skills improvement, attitude enhancement, and estimated numbers of patient consultations averaged 0.43, which together with the high intra-scale alpha reliability suggests that each of these five indices taps a different repertory of student performances that are inter-related but uniquely different from each other. Interestingly, it made a less-than-expected-difference whether these intercorrelations were tested for the enhanced (r = 0.44) or the control respondents (r = 0.33). Similarly, there was only slightly greater coherence among preceptors' assessments (r = 0.53) than among students' (r = 0.37). The general conclusion is the same in all instances – the five measures overlap to yield a reasonably generalized index of APPE training effectiveness which is significantly higher for the enhanced cohort than their classmates in either control setting.

### Instrument Refinement

Recognizing that completing a 70-item instrument was time-consuming and tedious for both students and preceptors, a subsequent phase of analysis tested whether a more streamlined instrument could be devised, and one which maximized the scales' differential abilities to discriminate between enhanced APPE and their traditional counterparts. Table 5 reports the item parameters for refined learning model scales when only the best-discriminating six items in each scale were included. Four, five and seven item versions were also tested but scale reliabilities, interscale differentiation, and scale sensitivity were all optimized with six items per scale. Selecting only those specific items from each learning model scale which most clearly distinguished between enhanced and control arms resulted in improved precision, such that all five refined scales discriminated significantly between the study's treatment arms whereas only three of the study's original scales were significant discriminators. The most common themes among items in the streamlined scales included: "patient follow-up," "documenting patient information," "providing pharmaceutical care-based care," "prioritizing drug-related problems," and "determining whether patients were managing their medications or had new questions or concerns," all of which reflect generally higher-level skills of pharmaceutical care practice rather than the more elementary learning tasks such as asking about patient expectations, collecting relevant information, etc. Streamlining the scales from 70 down to 25 items demonstrated the multiple advantages of brevity, greater precision, greater sensitivity to treatment improvements, all while maintaining absence of bias.

## Discussion

In general, the present study's results demonstrated that both students' and preceptors' outcomes favored the enhanced APPE in achieving the overall goals, although in slightly different fashion. Considering the combined student and preceptor outcomes on the full 70-item survey, the study's results suggested that the enhanced APPE resulted in about three times as many patient consultations as did the control experience, together with greater skills improvement, more favourable pharmaceutical care focused attitudes and an improved overall program impact of about 18%. Slight differences resulted when student and preceptor data was analyzed separately. For example, only the preceptor results suggested that students in the enhanced APPE were provided with a superior learning climate compared to students in the control APPE; and only the student results suggested that the enhanced APPE students experienced significantly greater attitude enhancement compare to their counterparts. Interestingly, the survey did not detect any difference in learning opportunity from the students' or preceptors' perspectives. When considering the 13 pharmaceutical care competency domains around which the APPE was structured from combined student and preceptor perspectives, all competencies favored the enhanced experience; and significantly so for all but two of the competencies (developing professional relationships with other health providers, physicians and participating in clinics, seminars, projects or presentations).

Past research has shown that curricular innovation is much more likely to be successful when instructors are provided with adequate opportunities to become familiar with the innovations and their rationale [20,21]. This applies equally well to preceptors asked to support changes to clinical placements such as those implemented in the enhanced APPE. Thus, while student accountabilities are spelled out in considerable detail in the SPEP manual given to students and preceptors in both control and enhanced APPE, preceptors in the enhanced APPE received additional education about how to foreground these expectations. This was accomplished by presenting the preceptors with suggestions on how to effectively set student expectations and facilitate student engagement in the available learning opportunities, as well as a discussion on each of the activities in which the students were asked to partake. Similarly, students were provided with a 5-day training that consisted of the Faculty discussing the APPE activities and expectations in detail with the students, giving the students the opportunity to spend time at their respective APPE sites prior to the start of their experience to familiarize themselves with their community pharmacy's processes and structures, initiate relationship and trust building with their preceptor and pharmacy staff, and allow their preceptor to assess the students' areas of strengths and weaknesses. Thus, the study instrumentation was built around procedures and accountability systems available equally to students and preceptors in both control and enhanced APPE. The non-difference in learning opportunity outcomes, but the significant difference in skills and attitudes indicates that the mere presence of opportunities is insufficient, but rather being expected to put them into practice with preceptors' guidance makes the enhanced model more successful than the traditional model as confirmed by the rich literature on situated learning in "communities of practice" [22]. In short, "we learn what we pay attention to," but when that attention is guided by explicit accountabilities, regular monitoring, and preceptor follow-up, the learning is more meaningful and better integrated [21-24].

### Pharmaceutical Care Competency Domains

In any learning venue, classroom or experiential, there is always the likelihood that some domains are apt to be better mastered than others; and there is instructionally diagnostic value in knowing which domains are successfully learned and which less so, and whether membership in the enhanced APPE cohort predicts learning gains in some domains more than others. Irrespective of the study arm, combined results from both students and preceptors showed that some of the pharmaceutical care competencies were better learned than others. For example, c*ollecting relevant patient information*, *questioning patients to determine their experiences with drug effectiveness or undesirable effects*, *determining whether they were adhering to their medication regimens *were all reasonably well achieved (mid 80%). In contrast, *participating in clinics, projects, presentations or seminars *was woefully underachieved (40%) irrespective of study arm. Between those two extremes, evidence shows clearly greater content mastery by students in the enhanced APPE; notably in *patient follow-up*, *integrating patient information*, *documenting for continuity of care*, and *establishing monitoring parameters*. For two domains (*developing professional relationships with physicians and other health care providers *and *participating in clinics, projects, presentations or seminars*), the control cohort fractionally outperformed the enhanced cohort but not significantly. In the present study, the finding that some competencies were better learned than others allows curriculum planners to focus on areas where more learning opportunities are required.

### Instrument Refinement

From the outset, it was evident that a 70-item survey was too long and oversampled the relevant content domains. Issues of frustration, inattention, and non-compliance are routine consequences of overlong surveys [25]. Nevertheless at the outset, it was unclear which items might prove to be the best indicators of the study's objectives, hence an initial oversampling was intentional for this proof-of-concept study. While there are many justifiable strategies for survey streamlining such as (1) item winnowing on the basis of face validity, (2) selecting only the half-dozen most reliable items in each scale, (3) scale reduction via factor analysis, or (4) multi-trait, multi-method matrix procedures, the overall objective in this study was to maximize the discrimination between enhanced and control APPE and to test for survey items which best accomplished that. Consequently, we examined the items within each learning model scale that demonstrated the highest enhanced versus control differentiation. In an effort to rebalance scale "lopsidedness" where some scales had as few as nine items while others had as many as 29, we examined results for four, five, six, and seven-item solutions for each of the 5-stage learning model scales. Table 5 shows the results for a six-item version (for each stage) which maximized the scales' sensitivities while retaining high alpha-reliabilities and holding scale bias to a minimum. Scale sensitivity (ability to discriminate between enhanced and control APPE) increased from 0.41 to 0.57 through judicious item selection. This refined combination of scale items allows accurate discrimination between enhanced settings and the two different control groups of 89.0% and distinguishes the enhanced from the combined control participants with an accuracy of 96.2%. As well, all five of the refined learning model scales become significantly sensitive to enhanced/control differences whereas in the original scales, only some were.

As before, the number of patient consultations is by far the best predictor of the enhanced APPE arm membership. In order of descending sensitivity within learning model scales, Table 6 shows these refined items with significance of Learning Model Scale sensitivity in brackets. In their refined forms, all five of the learning model scales became significant discriminators between enhanced and control APPE, and all but one was unbiased in terms of preceptor versus student differences. Only the estimated number of patient consultations again showed consistent overestimating by preceptors (p < 0.007) irrespective of enhanced vs. control membership. As Table 6 also shows, certain pharmaceutical care competencies such as documenting, providing follow-up, prioritizing drug-related problems, monitoring, etc., appear repeatedly – irrespective of whether they were phrased as Learning Opportunities, Skills Improvements, or Attitude-related. These are the pharmaceutical care practice aspects of patient consultations, a good indicator that direct interaction with patients is a most powerful learning tool.

**Table 6:** Refined scale composition with the six best-discriminating items per scale.

Number of patient Consulted: (F = 41.35, p < 0.000)*.	• Estimated number of consultations.
Learning Climate: (F = 3.88, p < 0.026).	• Preceptor encouraged patient follow-up. • SPEP was significant in stimulating PC delivery to patients. • Preceptor met with me on a regular basis to talk about patient issues. • Clerkship gave me opportunities to practice and improve my PC skills. • Preceptor identified activities for me to meet PC learning objectives. • Preceptor encouraged me to provide evidence and justify my recommendations.

Learning Opportunities: (F = 3.23, p < 0.046).	• Document patient information in forms usable by other pharmacists. • Provide follow-up (phone or in-person) using the monitoring plan. • Discuss monitoring parameters with patients regarding their therapies. • Determine whether patients are managing their medications or have questions. • Prioritize drug-related problems so that most relevant problems get addressed first. • Question patients to determine their experiences regarding drug effectiveness.

Skills Improvement: (F = 20.69, p < 0.000).	• Provide follow-up (phone or in-person) using the monitoring plan. • Integrate information for patients with multiple problems to identify drug-related problems. • Provide comprehensive PC to patients with fewer problems (4 or less). • Integrate patient information to identify actual/potential drug-related problems. • Prioritize drug-related problems so most relevant problems get addressed first. • Document patient information in forms to be used by other pharmacists/practitioners.

Attitude Enhancement: (F = 6.86, p < 0.002).	• Disagrees: Follow-up is too time-consuming. • Providing PC gives me a better understanding of patients' drug-related needs. • Agrees: I am comfortable with the process of providing PC. • Discussing monitoring parameters with patients is important. • Disagrees: Follow-up is too expensive. • Documenting care plans for continuity of care is important.

* F and p values indicate the significance with which each scale discriminated between enhanced and control experiences.

### Limitations

The study's interpretation and its generalization to other settings need to be viewed with certain cautions. The participant sample was both small and select, as befits a pilot study. Preceptors and students alike were volunteers and such self-selection may result in a Hawthorne-like upward drift in both interest and performance. Whether more mainstream participants (both preceptors and students) would demonstrate similarly favourable improvements between enhanced vs. control reports remains to be tested. The enhanced APPE intervention consisted of two components: preceptor education and student preparation prior to the start of the clerkship. One could reasonably question how much of the improved learning that was observed could have been the result of increased student preparation compared to preceptor training. The current study design and participant numbers do not allow a direct analysis of their reciprocal effects, and future investigation is planned. The study was retrospective in its conceptualization whereas pre/post designs coupled with enhanced/control conditions offer more explanatory power. The study's 70-item Scantron questionnaire presented some disadvantages. Questions and their responses were overprinted on about every third line of the Scantron bubbles resulting in alignment problems both in terms of printing and ease of reading. Future studies will ensure that respondents have the sole task of interpreting the questions and generating accurate responses while the researchers will accept the task of assigning scale numbers to conceptual categories even though it results in more laborious data entry. Also, 70 questions are undoubtedly too many resulting in respondent fatigue and loss of interest. Judicious combinations of reliability and discriminant analyses with larger and more representative samples will allow even further streamlining such that future questionnaires are shorter, more targeted and more focused, probably in the 20-to-30 item (and 6 or 7 domain) range of well-validated clinical learning instruments such as Stanford University's SFDP-26 or Cleveland Clinic's Teaching Effectiveness Instrument measures [26,27]. Finally, the learning of competencies was assessed, in part, by student self-report. It could be argued that it is in a student's best interest to report that learning has taken place, and that this motivation might be greater after a 5-day intervention. It was reassuring, therefore, to discover that comparisons of the students' perceptions of their learning to those of the preceptors were not statistically different. It can also be argued that student self-report and, more generally, the opportunities for students to reflect on their own learning, are vital components of programs such as the ones presented here.

## Conclusion

In summary, the proof-of-concept examination showed that the enhanced arm generated higher overall results than did the control arms, with specifically higher scores in student views about patient consultations, skills improvement and attitudes toward pharmaceutical care practice. Similarly, preceptors reported increased preceptor supports, numbers of patient consultations, student skills improvement, and enhanced student attitudes. These study outcomes helped the SPEP faculty to secure funding from several additional community pharmacy chains within British Columbia to further develop, evaluate and expand the enhanced APPE to other preceptors and students in future years. Concurrent with development of the enhanced APPE's rationale and operational realities, an assessment instrument was developed, reviewed and refined which was closely keyed to the requirements and expectations of SPEP objectives. Also, the psychometric properties of the assessment survey were examined during this first iteration of the enhanced SPEP rotation and shown to be generally supportive of the program's conceptual underpinnings – both in terms of learning process and of pharmaceutical care content domains. Finally, scale refinement analyses pointed to the need for briefer and more precise scales of clerkship learning and experience.

## Abbreviations

The following abbreviations were used but not defined in the text: SPSS, Statistical Package for the Social Sciences, ANOVA, Analysis of Variance

## Competing interests

The author(s) declare that they have no competing interests.

## Authors' contributions

RK conceived the study; participated in the design and implementation of the study and survey; participated in the acquisition of the data and interpretation of the data; and drafted and revised the manuscript. GP participated in the conception and design of the study and survey, participated in interpretation of the data and revision of the manuscript. JBC conducted data analysis and participated in the interpretation of the data and drafting and revising of the manuscript. All authors read and approved the final manuscript.

## Pre-publication history

The pre-publication history for this paper can be accessed here:


